# Structure of the intact ATM/Tel1 kinase

**DOI:** 10.1038/ncomms11655

**Published:** 2016-05-27

**Authors:** Xuejuan Wang, Huanyu Chu, Mengjuan Lv, Zhihui Zhang, Shuwan Qiu, Haiyan Liu, Xuetong Shen, Weiwu Wang, Gang Cai

**Affiliations:** 1 Key Laboratory of Agricultural and Environmental Microbiology, Ministry of Agriculture, College of Life Sciences, Nanjing Agricultural University, Nanjing 210095, China; 2 School of Life Sciences, University of Science and Technology of China, Anhui 230027, China; 3 Department of Epigenetics and Molecular Carcinogenesis, University of Texas M.D. Anderson Cancer Center, Smithville, Texas 78957, USA; 4 Hefei National Laboratory for Physical Sciences at the Microscale, Center for Integrative Imaging, Anhui 230027, China; 5 Center for Biomedical Engineering, University of Science and Technology of China, Anhui 230027, China

## Abstract

The ataxia-telangiectasia mutated (ATM) protein is an apical kinase that orchestrates the multifaceted DNA-damage response. Normally, ATM kinase is in an inactive, homodimer form and is transformed into monomers upon activation. Besides a conserved kinase domain at the C terminus, ATM contains three other structural modules, referred to as FAT, FATC and N-terminal helical solenoid. Here we report the first cryo-EM structure of ATM kinase, which is an intact homodimeric ATM/Tel1 from *Schizosaccharomyces pombe*. We show that two monomers directly contact head-to-head through the FAT and kinase domains. The tandem N-terminal helical solenoid tightly packs against the FAT and kinase domains. The structure suggests that ATM/Tel1 dimer interface and the consecutive HEAT repeats inhibit the binding of kinase substrates and regulators by steric hindrance. Our study provides a structural framework for understanding the mechanisms of ATM/Tel1 regulation as well as the development of new therapeutic agents.

## Abstract (web summary)

The ataxia-telangiectasia mutated (ATM) protein kinase is a key player in the response to DNA damage and is essential for the maintenance of cellular homeostasis. Here the authors describe the cryo-EM structure of ATM/Tel1 from fission yeast that suggests how dimerization regulates its activity.

## Graphical abstract

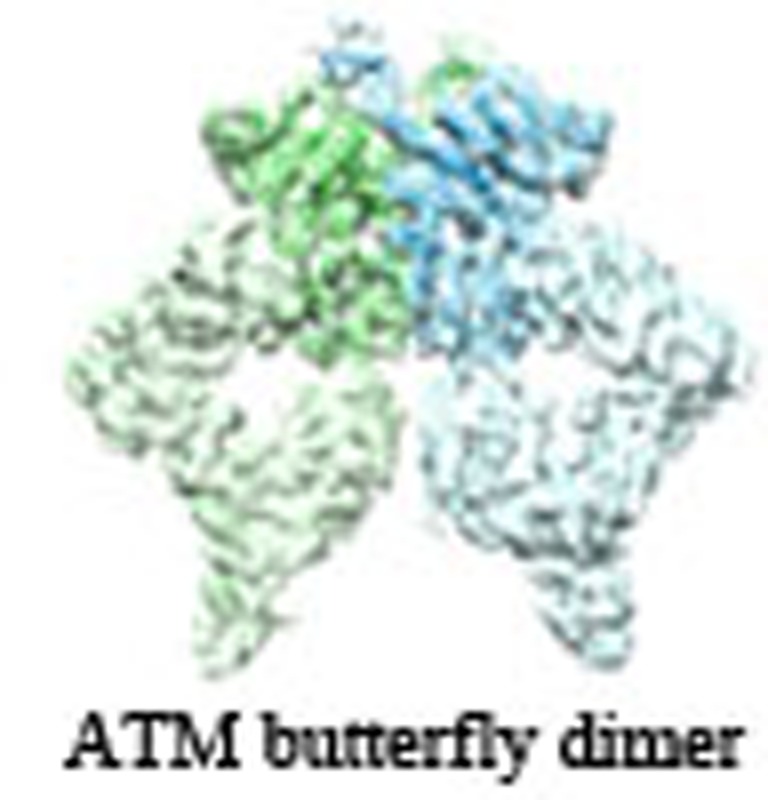

The genome is constantly under assault by environmental exposure to irradiation, chemical agents, ultraviolet light, as well as endogenous agents such as free radicals generated during normal metabolic processes. DNA double-strand breaks (DSBs) are considered to be the most deleterious DNA damage, which can directly lead to cell death if not repaired. When repaired incorrectly, DSBs can give rise to mutations and chromosomal rearrangements, leading to cancer in multicellular organisms^1^. The ataxia-telangiectasia mutated (ATM) protein is the apical kinase to trigger DNA-damage signalling required for the maintenance of genomic stability on DSB formations. Once activated, ATM phosphorylates a multitude (more than 700) of substrates^2^ such as p53 and checkpoint kinase Chk2, which are involved in cell-cycle control, DNA repair, cell survival and other cellular processes^2^,^3^. These ATM-dependent phosphorylation events are essential to arrest cell-cycle progression to allow DNA repair or to induce apoptotic cell death^4^. Consistently, ATM deficiency correlates with hypersensitivity to DNA-damaging agents, and in human ATM deficiency leads to ataxia telangiectasia (AT), a genetic disorder that is characterized by premature aging, cerebellar neuropathy, immunodeficiency and predisposition to cancer. Besides the role as an apical activator of the DNA-damage response for DSBs, ATM has also been suggested to be a versatile kinase involved in response to various genotoxic stresses and in diverse aspects of cellular homeostasis^3^.

The ATM protein, a large serine–threonine protein kinase (>300 kDa), belongs to the evolutionarily conserved phosphatidylinositol-3-kinase-related protein kinase (PIKK) family. There are six members in the family; each plays pivotal roles in controlling cellular homeostasis, including DNA-damage response (ATM, ATR and DNA-PKcs), cell growth (mTOR), mRNA decay (SMG1) and transcriptional regulation (TRRAP)^5^. The PIKKs contain a highly conserved C-terminal FAT/kinase/FATC domain architecture^6^,^7^ and an extended N-terminal helical solenoid of lower sequence similarity^8^,^9^. Owing to their important and diverse functions, sophisticated regulatory mechanisms are required to control the kinase activities of the PIKKs to adapt to particular pathways^10^. Under normal conditions, ATM kinase in cells is inactive and in the form of homodimers. Upon DSB formation, ATM homodimers quickly undergo intermolecular autophosphorylation and are transformed into monomers to fully activate the kinase activity^11^. Given the central roles of ATM in genome integrity and human diseases, it is essential to understand the mechanisms of ATM regulation. In particular, molecular and structural insights into ATM are critical to facilitate the design of therapeutic agents targeting the ATM/Tel1 kinase^4^,^5^. However, because of the large size (300∼500 kDa) and structural complexity, obtaining the high-resolution structure of PIKKs has remained a challenge. At present, except for the crystal structure of mTOR catalytic domain^12^, a 6.6-Å crystal structure of DNA-PKcs is the only model of a near-full-length PIKK^8^. Several EM structures were published, but the majority was determined under negative staining, which suffer from deformation artefacts induced by particle dehydration and distortion. Furthermore, the resolution of the structure is too low to even allow reliable docking of the crystal structures of the catalytic domain^13^.

In the fission yeast *Schizosaccharomyces pombe*, ATM is encoded by *Tel1* (telomere maintenance 1) gene^14^. The ATM/Tel1 kinase from *S. pombe* is one of the largest proteins, composed of 2,812 amino-acid residues with a molecular weight of 327 kDa. Cryo-EM is uniquely suited for structural analysis for such large and complex protein assembly. Here we report the first cryo-EM structure of the intact ATM kinase, which is in a non-covalent homodimer state. The 8.7-Å structure illuminated the intricate interface of ATM/Tel1 kinase homodimer and the unusual winding tertiary structure of the consecutively stacked N-terminal helical solenoid, as well as the distinct conformation of the N-terminal helical solenoid tightly packing against the FAT and kinase domains. These novel structural insights suggest that the dimer interface and the N-terminal helical solenoid could potentially allow tight regulation of the kinase activity by redundantly regulating the bindings of substrates and regulators. The detailed structural analysis on ATM/Tel1 also improves our understanding of structural organization and functional properties of other PIKKs. Moreover, this information could be used to develop specific inhibitors to lock the ATM in the inactive dimer state as potential radiosensitizers for cancer radiotherapy.

## Results

### Biochemical purification of ATM/Tel1 kinase

**Figure 1:**
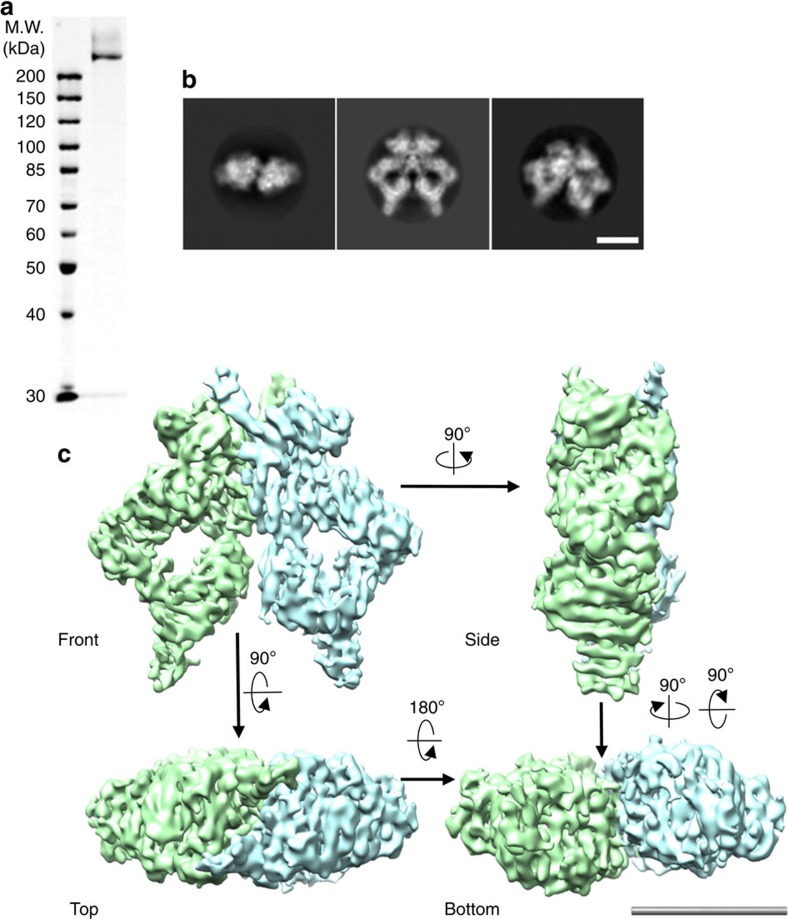
Purification and cryo-EM structure of intact ATM/Tel1 homodimer. (**a**) SDS–PAGE analysis of the intact ATM/Tel1 kinase endogenously purified from the *S. pombe*. (**b**) Three representative 2D class averages of ATM/Tel1 kinase. Scale bar, 100 Å. (**c**) The structure of ATM/Tel1 homodimer at 8.7 Å resolution, colour-coded by monomer assignment. Scale bar, 100 Å.

We have established an efficient purification procedure to acquire endogenous ATM/Tel1 directly from the yeast cells, which involves ammonium sulfate precipitation to enrich the ATM/Tel1-containing fraction and one-step affinity chromatography purification using IgG column. To further remove minor contaminants, an ion exchange Mono S column was employed. The procedure yielded highly homogeneous ATM/Tel1 kinase that is uniform in composition using SDS–PAGE analysis (Fig. 1a) and is satisfying for cryo-EM analysis. The particles observed with EM appeared well-preserved under negative stain and were similar to each other in size and overall shape (Supplementary Fig. 1a).

### Cryo-EM structure of homodimeric ATM/Tel1 kinase

We first performed negative-staining EM analysis to obtain the initial model for cryo-EM refinement using Random Conical Tilt (RCT) method (Supplementary Fig. 1b). Then, we determined the cryo-EM structure of the homodimeric ATM/Tel1 kinase. Consistent with the biochemical homogeneity of our ATM preparation as demonstrated by SDS–PAGE, cryo-EM images of the ATM/Tel1 particles preserved in vitrified ice (Supplementary Fig. 2a) are mostly homogeneous and are readily picked from the EM micrographs (Supplementary Fig. 2b). Through rigorous two-dimensional (2D; Fig. 1b and Supplementary Fig. 3a) and three-dimensional (3D) classifications using the RCT reconstruction of ATM/Tel1 under stain (low-pass-filtered to 60 Å) as an initial model, ∼32% particles are stably classified into a highly homogeneous class (Supplementary Fig. 3b). To refine this subset to high resolution in a fully automated manner, the 3D auto-refine procedure in RELION was used, resulting in a cryo-EM reconstruction of ATM/Tel1 kinase (Fig. 1c). The resolution was estimated objectively at 8.7 Å (Supplementary Figs 3c and 4b), using the gold-standard Fourier Shell Correlation (FSC) calculations.

The intact ATM/Tel1 kinase directly purified from the yeast cells is a homodimer, and the dimeric architecture resembles a butterfly stretching out its wings (Fig. 1c). Two ATM/Tel1 molecules are juxtaposed in a side-by-side manner, with their top region interacting with each other. This high-resolution structure has allowed us to dissect the structural mechanisms of ATM/Tel1 at an unprecedented level. Particularly noteworthy is that the majority of the ATM/Tel1 domains are composed of helix-turn-helix repeats, with the α-helix being the dominant secondary structure (Supplementary Fig. 4c).

### ATM/Tel1 structural modules

**Figure 2:**
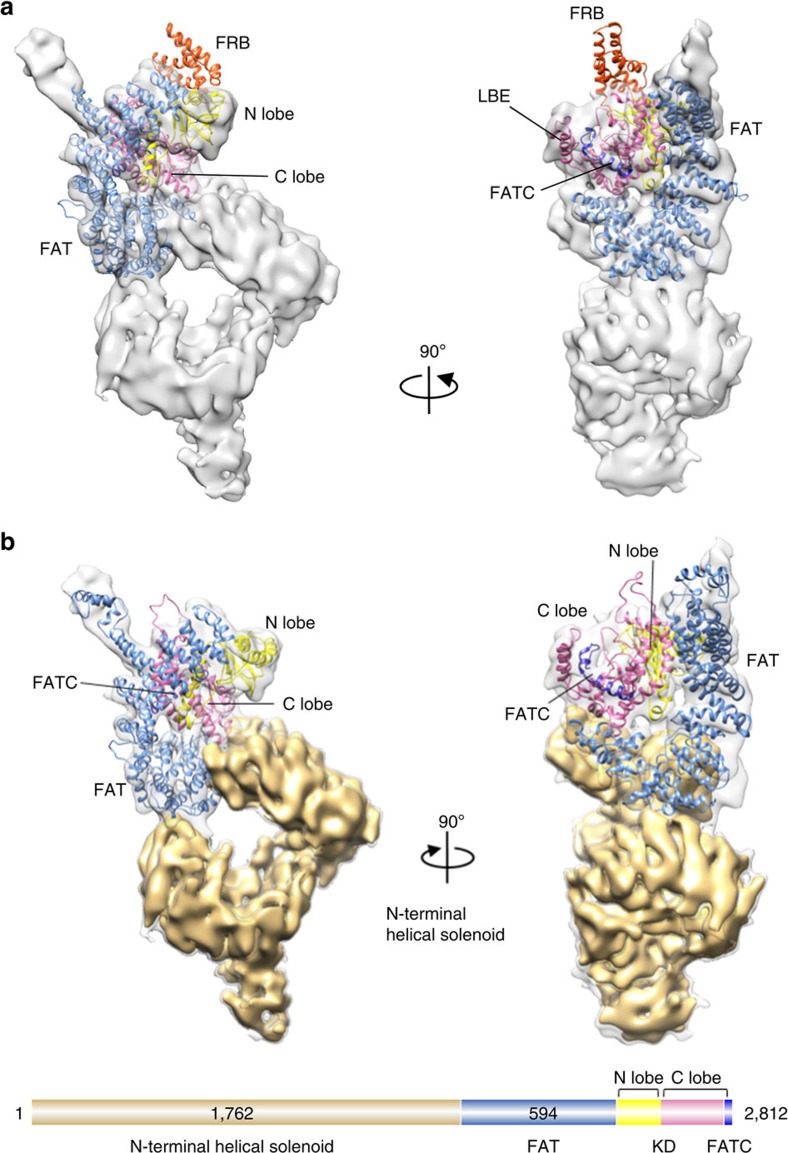
Modular architecture of ATM/Tel1 kinase. (**a**) The structure of ATM/Tel1 monomer fitted with crystal structure of mTOR catalytic core (PDB ID: 4JSV)^12^ as a rigid body. (**b**) Model of the ATM/Tel1 monomer, colour-coded by domain assignment (top). The EM structure of ATM/Tel1 monomer is fitted with the C-terminal ATM/Tel1 homology modelling model (residues: 1,786–2,812) based on the crystal structure of mTOR catalytic core (PDB ID: 4JSV). Domain organization of ATM/Tel1 kinase (bottom). The number of residues of N-terminal helical solenoid and FAT domains are indicated.

The unprecedented details apparent in the ATM/Tel1 structure prompted us to delineate its modular architecture. All the PIKKs contain a highly conserved C-terminal FAT/kinase/FATC domain architecture^6^,^7^. Consistently, the crystal structure of mTOR catalytic core (PDB ID: 4JSV)^12^ could be unambiguously fitted in the Head region of the ATM/Tel1 kinase cryo-EM map without flexible fitting (Fig. 2a and Supplementary Fig. 5). The only exception is that the unique FRB domain insertion of mTOR could not be fitted and sticks out from the N-lobe of the kinase domain. A general correspondence between the crystal structure of mTOR catalytic core and the cryo-EM structure of the Head domain of the ATM/Tel1 corroborates the high conservation of C terminus of PIKKs.

On the basis of the crystal structure of mTOR kinase (PDB ID: 4JSV), we modelled the C-terminal 1,027 residues of ATM/Tel1 through homology modelling followed by Molecular Dynamics Flexible Fitting (MDFF)^15^ to optimize the fitting into the EM map. The kinase domain of ATM/Tel1 kinase has a typical bi-lobed structure, and the active site is deeply recessed inside a cleft, which is generally in the intrinsically active conformation^5^,^12^,^13^. The FAT domain intimately wraps around ∼50% of the kinase domain, which has been suggested to regulate the binding of kinase substrates or regulators^5^ (Fig. 2b and Supplementary Fig. 6). In addition, adopting a highly sinuous superhelical structure, the N-terminal helical solenoid formed two arms, cradling the catalytic core on top. These two observations are consistent with the model that the intrinsically active conformation of the kinase domain and restricted access of the active site are general features of the PIKKs^10^,^12^.

### ATM/Tel1 dimerization interface

The dimer architecture of PIKKs is conserved and dimerization has been implicated in the proper functions of several PIKKs, such as ATM, DNA-PKcs and mTOR^10^,^13^. Similar to PI3K in its auto-inhibited basal state, each subunit of the dimer likely participates in blocking the activity of the other subunit^4^. It has been suggested that inactive ATM exists as a non-covalent homodimer in cells, but is rapidly activated and converted into monomer on sensing the DNA DSBs^11^,^14^,^16^. Owing to the lack of the structural information, the detailed mechanisms for PIKK dimerization and activation remain largely unknown^4^.

**Figure 3:**
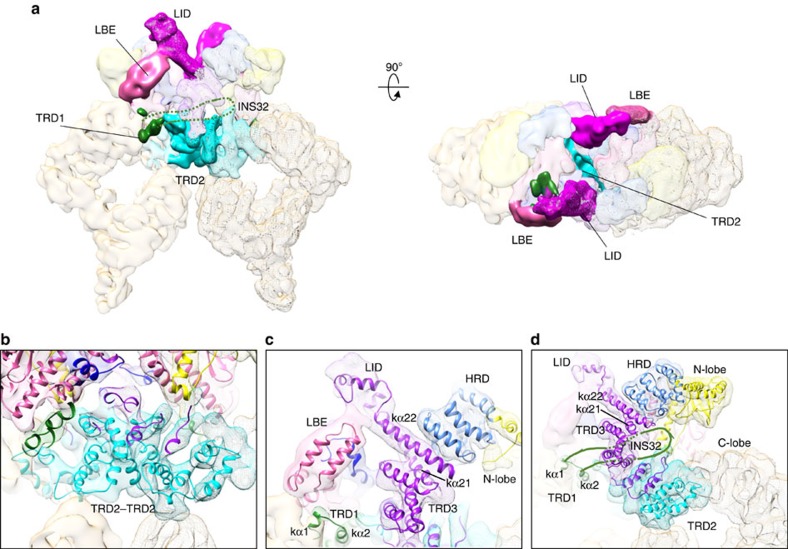
Three prominent dimer interfaces of ATM/Tel1 kinase. (**a**) EM models of ATM/Tel1 dimer highlighting the following three dimer interfaces. Two monomers of ATM/Tel1 are displayed as surface and mesh. Domains are colour-coded as in Supplementary Fig. 6. (**b**) Enlarged view of the TRD2–TRD2 interface. (**c**) Enlarged view of the LID–LBE interface. (**d**) Enlarged view of the putative INS32–AS (active site) interface in the ATM kinase from higher eukaryotes.

The structure of the ATM/Tel1 homodimer provides unprecedented structural insight into the dimer architecture of PIKKs and the mechanisms of ATM's auto-inhibition in the dimeric state (Fig. 3a).

The cryo-EM map fitted with the homology model of the C-terminal of ATM/Tel1 illuminated that the dimer interface is mainly centred on the TRD2 regions of the FAT domains between the two monomers, which constitutes the largest part of this interface (Fig. 3b). The arrangement of only helices close to the dimer interface generates a large curved surface across the TRD2 domains (Supplementary Fig. 7). It is tempting to propose that the dimer interface observed in the ATM/Tel1 kinase is conserved among the PIKKs. In this case, several mutations in mTOR that lead its activation (W1449, A1459, L1460, C1483, and A1577) are also localized in the TRD2 domain^12^, which is consistent with the notion that dimerization interface of ATM/Tel1 across the TRD2 domains seems critical to stabilize the dimeric state. Thus, the cryo-EM structure of ATM/Tel1 suggests that these mutations in the TRD2 domain likely destabilize the dimer interface of mTOR.

All PIKKs also contain a LST8-binding element (LBE)-like insertion in the C-lobe of the kinase domain, which is potentially involved in interactions with PIKK regulators^10^,^12^,^13^. For example, the LBE domain of mTOR directly interacts with its regulator mLST8 (ref. 5). In the dimer structure of ATM/Tel1, a rod-shaped protrusion density leans on the LBE domain, which corresponds to the linker region of the α21 and α22 of the TRD3 domain (Fig. 3c). Sequence analysis suggests that the secondary structure of the linker region is mainly α-helix, which is consistent with the observed rod-shaped density of the protrusion. Intriguingly, the region of ATM/Tel1 interacting with the conserved LBE-like domain is also highly conserved (Supplementary Fig. 8a). Therefore, we name the protrusion as LBE-interacting domain (LID). In the dimeric state, the LID from one ATM monomer contacts the LBE of the other molecule, possibly blocking a putative ATM regulator binding to LBE, and thus could inhibit the activation of ATM/Tel1 kinase. Consistently, in the mTOR dimer model derived through fitting the crystal structure of mTOR catalytic core into the cryo-EM structure of ATM/Tel1 dimer, the LID domain would directly block mLST8 binding to the LBE domain^12^ (Supplementary Fig. 9). Such an intermolecular LID–LBE interaction of ATM/Tel1 constitutes the second dimer interface, which could play a critical role in inhibiting the activation of ATM/Tel1 (probably in other PIKKs as well) by blocking the binding of regulators (Fig. 3c).

In higher eukaryotes, intermolecular autophosphorylation of Ser1981 has been proposed to induce dimer dissociation and it initiates ATM kinase activity^11^. Ser1981 lies in the middle of a 32-amino-acid insertion between the α1 and α2 of the TRD1 region of the FAT domain. We named the 32-amino-acid insertion INS32 (Supplementary Fig. 8b). Insertion INS32 and the transphosphorylation of Ser1981 within the insertion are highly conserved in the ATM of higher eukaryotes, but are absent in yeast ATM/Tel1 (ref. 4). This part of the FAT domain plays important roles not only in ATM (S1981) but also in ATR (T1989)^17^ and DNA-PKcs (2,023–2,056 and 2,609–2,647; ref. 18). The disordered INS32 probably intertwines with the active site of another molecule, which enables the kinase domain to bind to the region surrounding Ser1981 for its transphosphorylation^11^. In addition, it could potentially restrict the substrate access (Fig. 3d). In this regard, one effect of the Ser1981 autophosphorylation in higher eukaryotes could be to induce the conformational changes of the INS32 to release its binding and inhibition of the active site.

### Structure of the N-terminal helical solenoid

**Figure 4:**
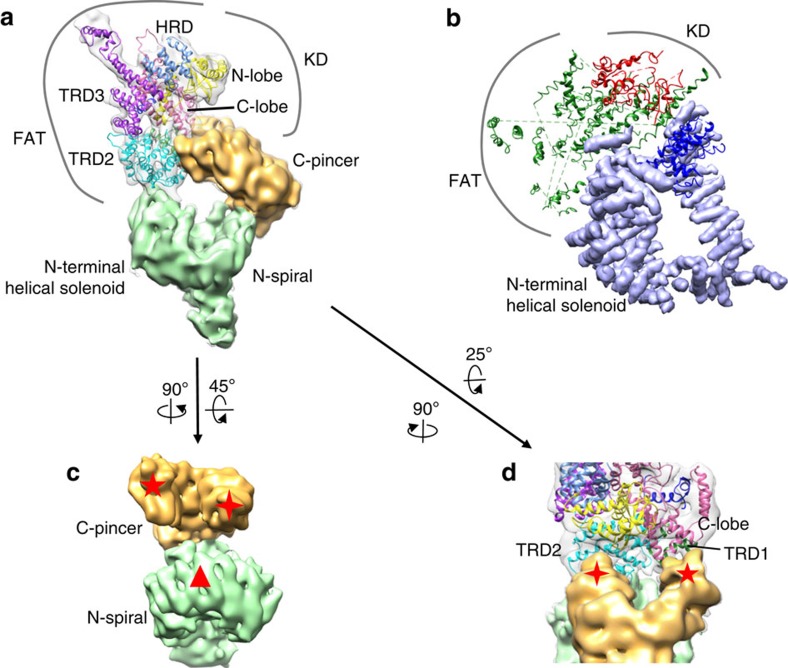
Overall architecture of the N-terminal helical solenoid. (**a**) Model of ATM/Tel1 monomer highlighting the two arms of the N-terminal helical solenoid: C-pincer and N-spiral are coloured as orange and green, respectively. (**b**) A model for DNA-PK (PDB ID: 3KGV)^8^ coloured by the FAT/kinase/N-terminal helical solenoid domains. (**c**) Three interfaces of the C-pincer and N-spiral interacting with the FAT and kinase domains. (**d**) Enlarged view of two interactions of the C-pincer with the FAT domain and C-lobe of kinase domain. The triangle shows the interface of N-spiral contacting with the TRD2 domain of the FAT region. The four-pointed star shows one tip of the C-pincer interacting with the TRD2 domain of the FAT region. The five-pointed star shows another tip of the C-pincer binding with TRD1 domain of the FAT region and C-lobe of the kinase domain.

The N terminus of all the PIKKs is composed entirely of HEAT repeats, forming a superhelix or α-solenoid, for which limited structural information is available^10^. The consecutively stacked N-terminal helical solenoid of ATM/Tel1 kinase yields an unusually winding tertiary structure with two arms. Therefore, we morphologically segment the N-terminal helical solenoid as C-pincer and N-spiral. In a minor population of the ATM/Tel1 particles, the bottom part of the N-terminal helical solenoid, corresponding to the N-spiral, could sway far from the dimer interface (data not shown). We thus deduce that the C-pincer of the N-terminal helical solenoid is covalently connected to the FAT domain of the ATM/Tel1 kinase (Fig. 4a).

Similar to the α-solenoids of DNA-PKcs^8^, the two arms of the N-terminal helical solenoid of ATM/Tel1 directly interact with both the FAT and kinase domains (Fig. 4a,b). In particular, one tip of the C-pincer binds to the TRD2 region of the FAT domain; another tightly interacts with both the C-lobe of the kinase domain and the TRD1 region of the FAT domain (Fig. 4c,d). The N-spiral packs against another region within the TRD2 domain (Fig. 4a,c). Therefore, the entire catalytic core of PIKKs appears tightly coupled to the N-terminal helical solenoid, consistent with the previous structural and functional finding that the N-terminal helical solenoid may serve as a scaffold^10^. Furthermore, the N-terminal helical solenoid also has been reported to be essential for binding proteins that are associated with the PIKKs to regulate their activities and cellular localizations^10^. In summary, our ATM/Tel1 structure has revealed the architecture of this highly important kinase, and provided novel mechanistic insights into how the N-terminal helical solenoid interact with FAT and kinase domains.

## Discussion

Since ATM is a central and versatile kinase involved in diverse aspects of cellular homeostasis^3^, it is critical to tightly regulate its kinase activity. Accordingly, it has been proposed that ATM is held inactive in cells as a homodimer and quickly transits from dimer into active monomer in response to DNA damage^11^. The intact ATM/Tel1 kinase directly purified from *S. pombe* grown under normal conditions is an inactive homodimer, which suggests that each molecule likely participates in blocking the activity of the other, locking the dimer in an auto-inhibited basal state^11^. Importantly, the dimeric architecture of PIKKs appears to be conserved^5^,^13^, which has been implicated in the proper functions of ATM^11^, DNA-PKcs^19^ and mTOR^20^.

The dimeric ATM/Tel1 structure suggests that each ATM monomer likely participates in inhibiting the activity of the other via at least three interfaces: TRD2–TRD2, LBE–LID and INS32–active site. The TRD2–TRD2 interaction surface constitutes the main scaffold of the dimer interface. Furthermore, the LBE–LID and INS32–active site interfaces could directly inhibit the binding of ATM regulators and block the substrate-binding site, respectively. It is conceivable that the dual blockage mechanism may also be utilized by other PIKK dimers. Moreover, not only with a scaffolding function, the N-terminal helical solenoid could also contribute to control the recruitment and delivery of specific substrates and regulators^10^. Through these multiple and sophisticated mechanisms, the kinase activity of ATM/Tel1 is strongly and redundantly auto-inhibited in the homodimeric state. The dimer–monomer transition of ATM/Tel1 may therefore function to uncover the substrate and regulator-binding domains and allow the formation of active complexes. Therefore, the homodimeric ATM/Tel1 structure offers a structural framework to understand the mechanisms of ATM kinase activation and its regulation.

ATM/Tel1 is the apical kinase to initiate the multifaceted DNA-damage responses to DNA DSBs. Owing to its pivotal roles in the regulation of genomic integrity, ATM has been a potentially viable therapeutic target^4^,^5^,^21^. On the basis of the structure of ATM/Tel1 dimer, specific ATM inhibitors could be developed to lock the ATM kinase into the inactive dimeric state. Moreover, conjugating such specific ATM inhibitors with coupling agents harboring high affinity to cancer cells would result in new therapeutic agents as radiosensitizers. By targeting tumour cells during the radiation therapy, radiosensitizers may selectively induce catastrophic genomic instability and cause lethality in cancer cells. Our dimeric ATM/Tel1 structure provides an initial molecular blueprint for the development of potential radiosensitizers for cancer radiotherapy. However, the resolution of the current EM structure of ATM is still too low to directly guide the design of such radiosensitizers. The major limiting factor in increasing the resolution of the ATM/Tel1 cryo-EM structure is the strong preferred orientations of the particles in the vitrified ice (Supplementary Fig. 4a). Further improving the cryo-EM reconstruction of ATM/Tel1 kinase through optimizing the coverage of spatial orientation will critically contribute to providing critical structural information required for the development of drugs targeting ATM.

## Methods

### Purification of Yeast ATM/Tel1

*S. pombe* stain CC5060 (*h–leu1-32 ura4-D18 kanMX6:nmt1:TAP-tel1*)^14^ was grown in YPD medium to the stationary phase. About 100 g cells were harvested, washed and resuspended in extraction buffer (50 mM HEPES (pH 7.6), 300 mM KOAc, 0.5 mM EDTA, 5 mM β-ME, 10% (v/v) glycerol, 0.1% (v/v) NP-40 and protease inhibitors), and a whole-cell extraction was prepared as previously described^22^. This whole-cell extract was selectively precipitated in 30–55% ammonium sulfate and resuspended using 1 × TEZ buffer (50 mM Tris-HCl (pH 7.5), 1 mM EDTA, 10 mM ZnCl_2_, 5 mM β-ME and protease inhibitors). After the suspension was clarified using centrifugation, the supernatant was incubated for 2 h at 4 °C with 1 ml of a 50% slurry of IgG-sepharose resin beads (GE Healthcare) that had been pre-equilibrated with 1 × TEZ plus 250 mM ammonium sulfate. After incubation, the beads were washed with 50 ml of 1 × TEZ plus 500 mM ammonium sulfate, followed by a second wash with 50 ml of 1 × TEZ plus 50 mM ammonium sulfate. After equilibration of the column with 1 × TEZ plus 100 mM ammonium sulfate (without protease inhibitors), 100 units of AcTEV protease (Invitrogen) was added to the resin beads and incubated overnight at 4 °C. The ATM/Tel1 fraction was then eluted with three column volumes of 1 × TEZ plus 100 mM ammonium sulfate and 10% glycerol was added, and the resulting aliquot was snap-frozen in liquid nitrogen and temporarily stored at −80 °C. For the next purification step, the IgG eluate fractions were thawed in ice and applied on a Mono S column (GE Healthcare) in Q100 buffer (100 mM ammonium sulfate, 50 mM Tris, pH 7.6, 10% v/v glycerol, 1 mM EDTA, 10 μM ZnSO_4_, 0.02% NP-40 and 10 mM β-ME) and was resolved over a 100–1,000-mM ammonium sulfate gradient. The ATM/Tel1 elution was flash-frozen in liquid nitrogen, and was analysed using SDS–PAGE and EM examination.

### EM sample preparation and RCT reconstruction

The Mono S peak fraction was diluted eight times (20 mM HEPES (pH 8.0), 40 mM KOAc, 5 mM MgCl_2_, 0.1% trehalose, 2 mM dithiothreitol (DTT) and 0.01% NP-40) and was applied to a freshly glow-discharged carbon-coated 400-mesh Cu EM specimen grid, and then preserved by staining with 0.75% (w/w) uranyl formate solution. Images were recorded at a magnification of × 62,000 on a 4,096 × 4,096 CCD (charge-coupled device) detector (FEI Eagle) with a Tecnai F20 electron microscope (FEI) operating at an acceleration voltage of 200 kV. Images were recorded by using low-dose procedures at ∼0.6–0.8 μm under focus. Twofold pixel binning of the original CCD images resulted in a final pixel size of 3.54 Å per pixel.

The particles of the ATM/Tel1 kinase show a strongly preferred orientation on adsorption to amorphous carbon support films. To obtain the initial model for cryo-EM refinement, 3D reconstructions were calculated by using the RCT method^23^. Tilted (−55°) and untilted image pairs were obtained under low-dose conditions, and particles were selected using the TiltPicker programme^24^ and were montaged for interactive screening, yielding ∼11,000 tilt-pair images of the ATM/Tel1. We run iterative alternating rounds of supervised multireference alignment and classification as well as reference-free alignment to improve the homogeneity of the image classes. All the 3D reconstructions were calculated with SPIDER^25^ and SPARX^26^.

### Sample vitrification and cryo-EM data collection

Samples were diluted to a final concentration of 20–50 μg ml^−1^ (20 mM HEPES (pH 8.0), 40 mM KOAc, 5 mM MgCl_2_, 0.1% trehalose, 2 mM DTT and 0.01% NP-40), and 3 μl of aliquots was applied to freshly glow-discharged Quantifoil R2/1 grids coated with a second layer of thin carbon film. The grids were blotted for 3–4 s at 4 °C in 100% humility, and then plunged into liquid ethane using an FEI Vitrobot (FEI Company). Frozen grids were stored in liquid nitrogen. The grids were first loaded into a Gatan 626 cryo-holder and transferred to an FEI Tecnai TF20 electron microscope to check the quality of the sample vitrification. Then, the grids were transferred to Titan Krios equipped with a field emission source and were operated at 300 kV. Images were recorded on a Falcon 2 direct electron detector at a nominal magnification of × 59,000 with a defocus range of 2–4 μm, resulting in a calibrated sampling of 1.42 Å per pixel. The total accumulated dose rate was set to be 35 e^2^ per Å^2^ on the specimen, and the exposure time was 1.5 s. Each image was fractionated into 23 frames.

### Image processing

Frames were summed to a single micrograph for subsequent processing using motion-correction procedure described by Li *et al*.^27^. Contrast transfer function (CTF) parameters and defocus values for each micrograph were determined by using CTFFIND3 (ref. 28). A semi-automated procedure in RELION^29^ was used to pick particles. Overall, 57,435 particles were picked after carefully sorting and cleaning. 2D and 3D classification and auto-refinement were performed using RELION. The initial RCT model was low-pass-filtered to 60 Å and was used as the starting model for the 3D classification. 3D refinement used gold-standard FSC calculations to avoid overfitting, and reported resolutions are based on the FSC 0.143 criterion. The 17,894 ‘good' particles' group was used to polish the refinement. A soft mask was used during the auto-refinement procedure to improve the resolution, and postprocessing was carried out by ‘postprocess procedure' in Relion using a provided B-factor −400 and a soft spherical mask (with a 5-pixel fall-off). The final resolution reported was estimated from the masking-effect-corrected FSC curve using the FSC 0.143 criterion. All the 3D structures were displayed using Chimera^30^.

### Model building and simulation

A homology model of the C-terminal 1,027 residues of ATM/Tel1 was built with Modeller^31^ using the crystal structure of mTOR catalytic core (PBD ID: 4JSV)^12^ as a template. Missing and unaligned residues, including residues 2,055D–2,080N, 2,185F–2,231I, 2,393L–2,433K and 2,721L–2,754P, were rebuilt with I-TASSER^32^. This model was used as input for MDFF^15^ to fit the density map. All the simulations were prepared using VMD^33^ and were performed using NAMD^34^. The mdff package was used for the density-fitting-related preparations. The griddx command was used to convert the density map to MDFF potential. The CHARMM27 force field was used for the molecular dynamics. The ssrestrains command was used to enforce restraints on secondary structure elements. MDFF modelling was carried out in two consecutive stages, as outlined in the MDFF tutorial. In the first stage, a short 200-step minimization was carried out to optimize bond geometries and remove clashes. Then, a 1-ns simulation was performed using a low-density scaling factor of 0.3 (gscale=0.3). In the second stage, a 200-ps simulation using a higher-density scaling factor of 5 (gscale=5) followed the simulation in the first stage.

### Data availability statement

The 8.7-Å cryo-EM structure of ATM/Tel1 kinase from *S. pombe* is available from the EMDB with accession code EMD-6399. The authors declare that all other data supporting the findings of this study are available within the article and its Supplementary Information Files.

## Additional information

**How to cite this article**: Wang, X. *et al*. Structure of the intact ATM/Tel1 kinase. *Nat. Commun.* 7:11655 doi: 10.1038/ncomms11655 (2016).

## Supplementary Material

Supplementary Information Supplementary Figures 1 - 9 and Supplementary References
