# Supplementary material for: Structure of the intact ATM/Tel1 kinase
Source: Nat Commun. 2016 May 27;7:11655. doi: 10.1038/ncomms11655 (PMC4894967; doi:10.1038/ncomms11655)
Supplement: Supplementary Information — Supplementary Figures 1 - 9 and Supplementary References [file ncomms11655-s1.pdf]

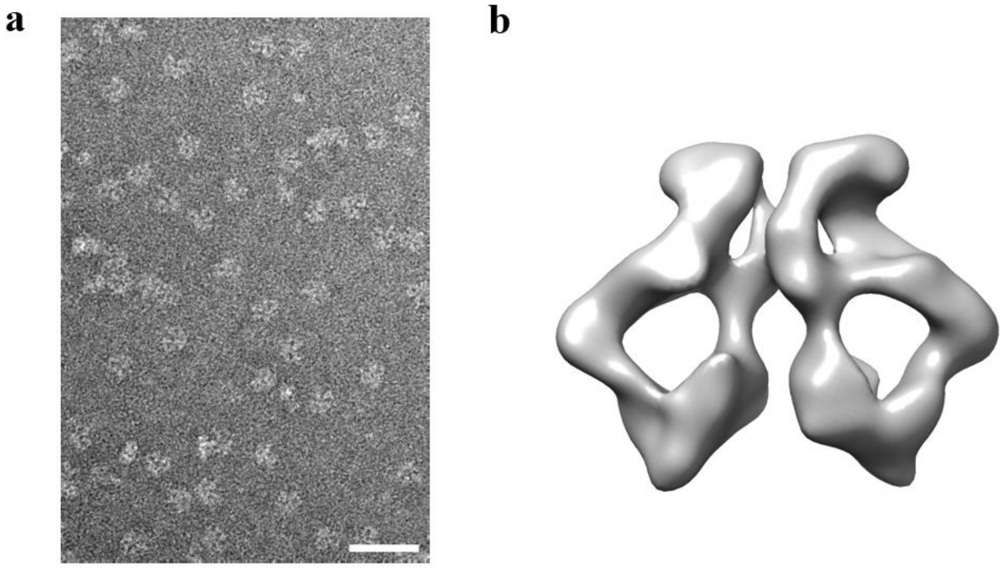

Supplementary Figure 1 | Initial model of ATM/Tel1 kinase. (**a**) A typical micrograph of ATM/Tel1 particles preserved in negative stain. Scale bar, 50 Å. (**b**) Initial model for cryo-EM refinement calculated by using the random conical tilt (RCT) method [1].

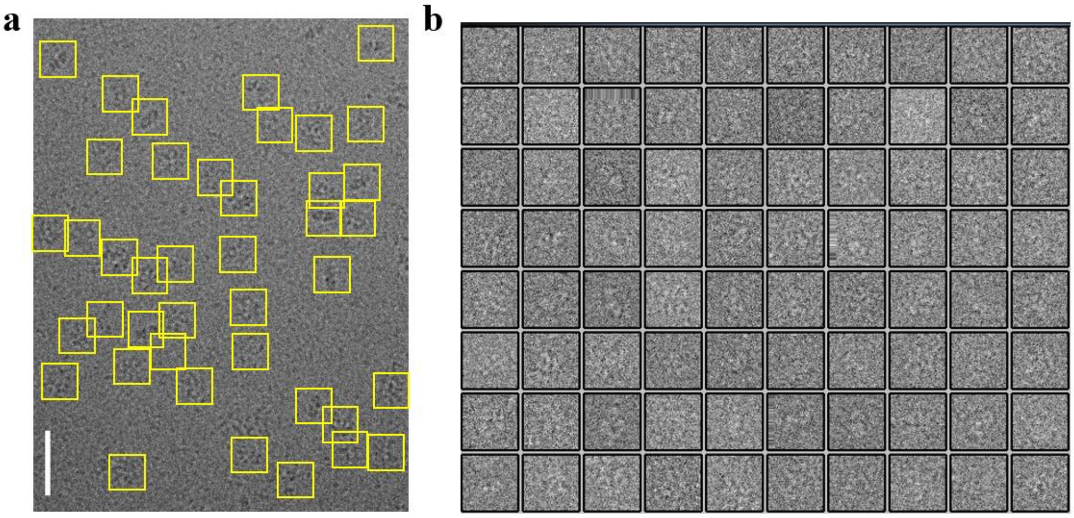

Supplementary Figure 2 | Cryo-EM analysis of ATM/Tel1 kinase.

(**a**) A typical micrograph of ATM/Tel1 particles preserved in vitrified ice. The positions of the particles are identified by yellow squares. Scale bar, 50 Å. (**b**) The typical single particles.

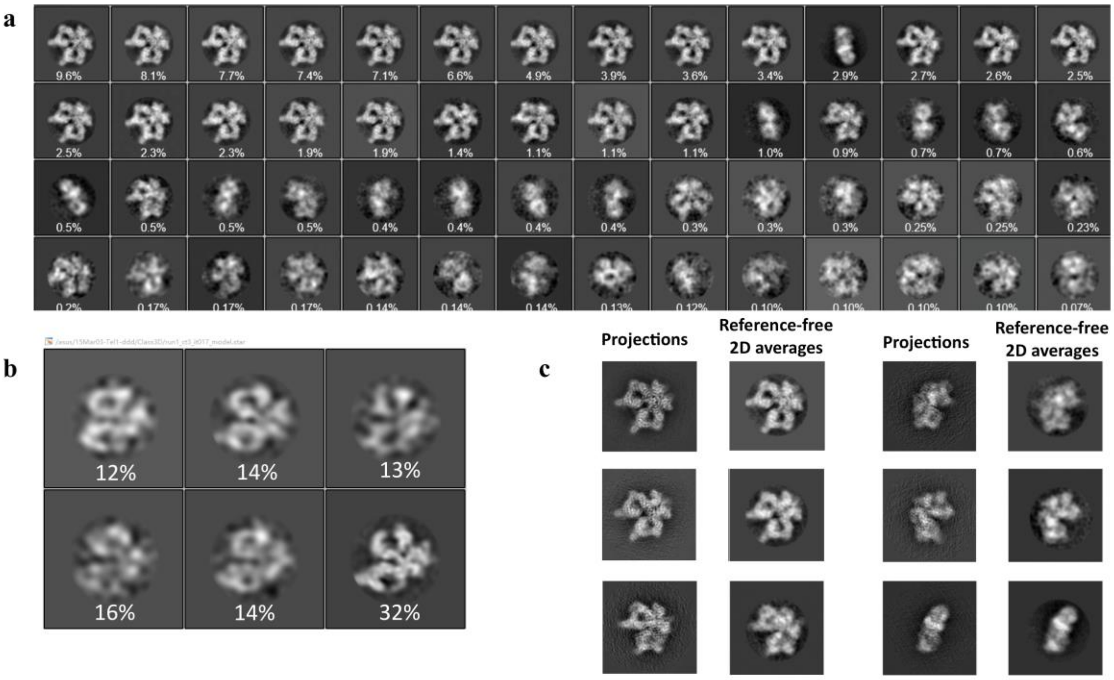

Supplementary Figure 3 | Cryo-EM reconstruction of ATM/Tel1 kinase. (**a**) Typical 2D class averages obtained after reference-free alignment and classification of images of ATM/Tel1 particles preserved in vitrified ice. The percentages of each class are indicated below the averages. (**b**) Six reconstructions calculated by 3D classification using Relion [2]. The last class was used for further refinement. (**c**) Close correspondence between projections of the ATM/Tel1 cryo-EM structure and 2D reference-free class averages.

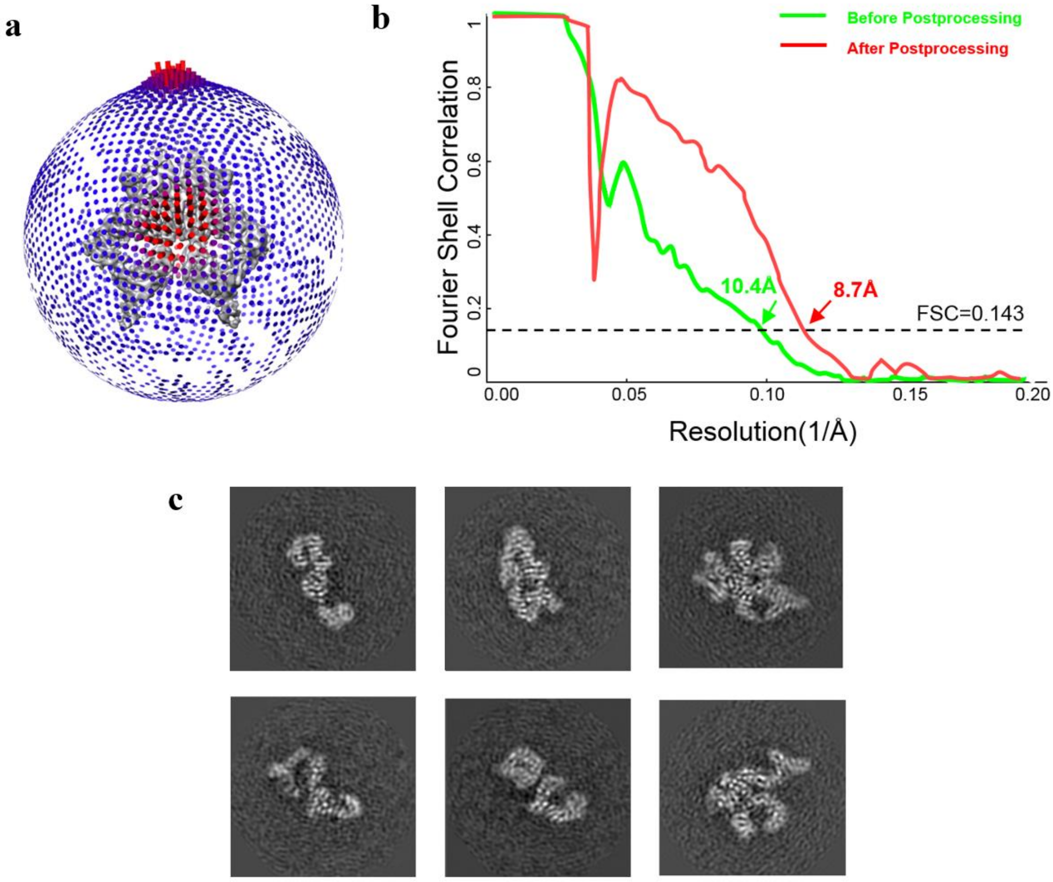

Supplementary Figure 4 | Cryo-EM analysis of the ATM/Tel1 kinase. (**a**) Angular distribution for the final reconstruction of the ATM/Tel1 kinase. Each cylinder represents one view and the height of the cylinder is proportional to the number of particles for that view. (**b**) FSC curve for the cryo-EM density map according to the gold-standard criterion. The final resolution is 8.7 Å. (**c**) Six figures clearly show alpha helices can be resolved from the directions from several views (including the top, front and rare side views).

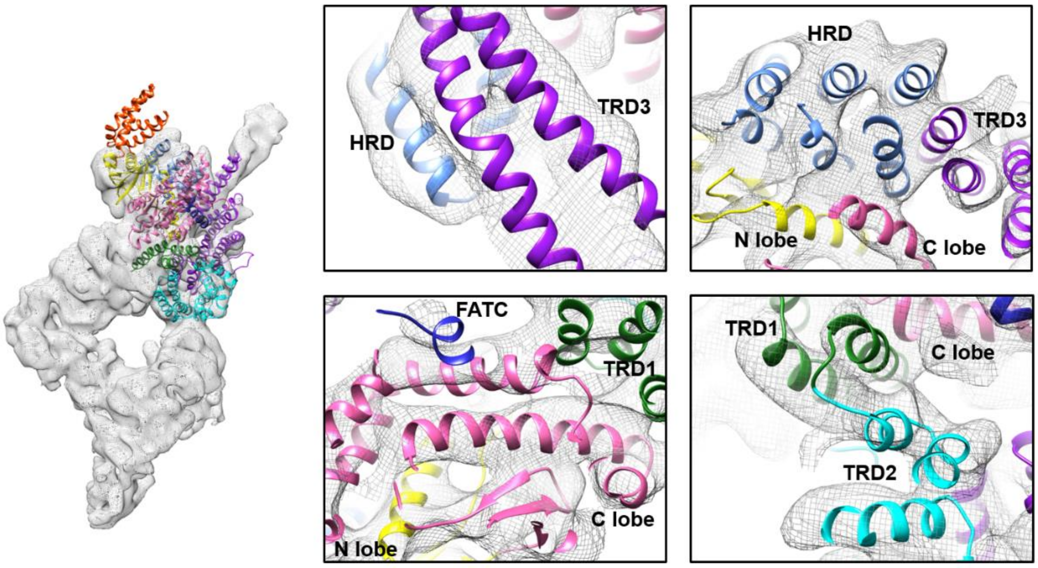

Supplementary Figure 5 | Rigid-body fitting the crystal structure of mTOR catalytic core (left, PDB ID: 4JSV) [3]. Four representative views showing the mTOR structure can be well fitted into the cryo-EM map of ATM/Tel1 kinase (right).

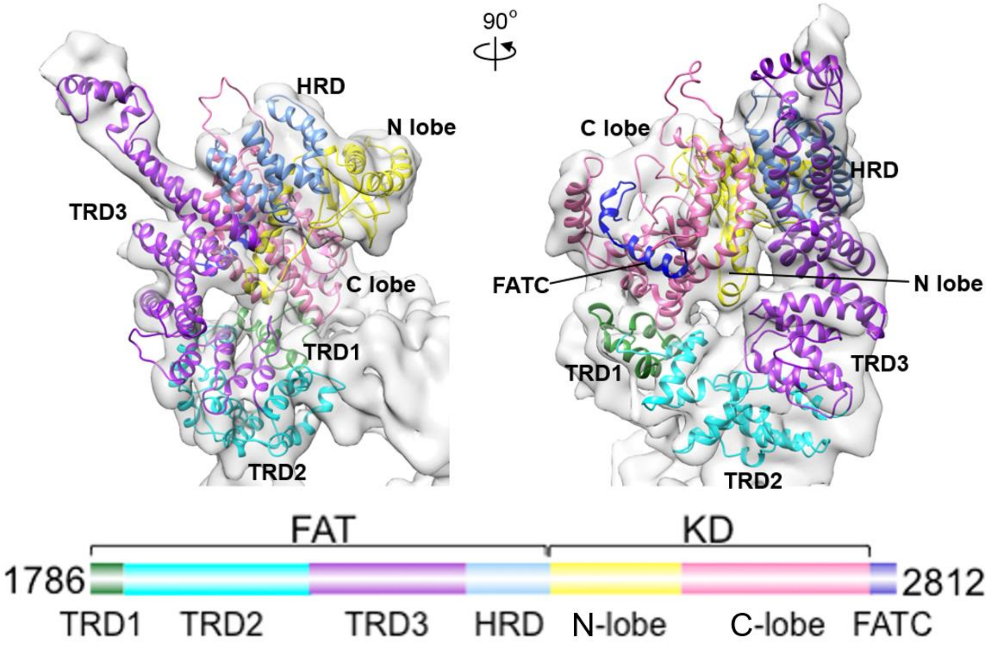

Supplementary Figure 6 | Enlarged view of the structure of the catalytic core of ATM/Tel1 model (top). Detailed domain diagram of ATM/Tel1 catalytic core (bottom).

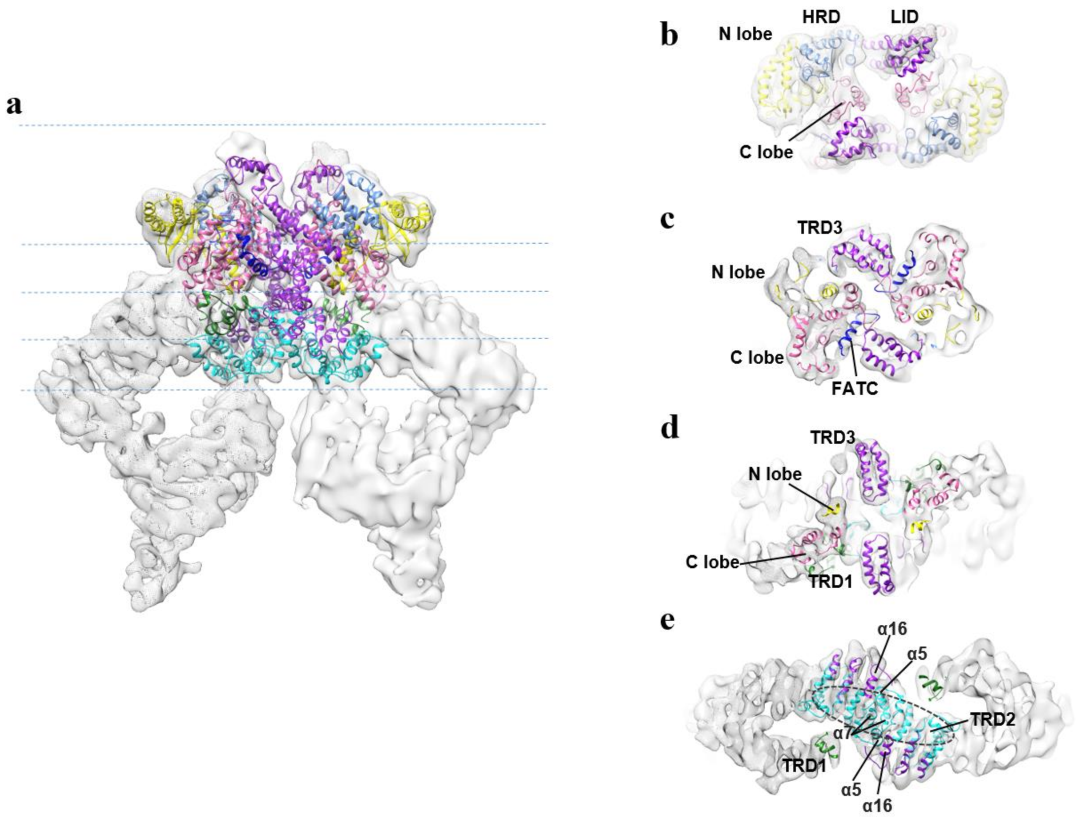

Supplementary Figure 7 | Details of the ATM/Tel1 dimer interface.

(**a**) Front view of ATM/Tel1 kinase fitted by the homology-modeling model (residue: 1786-2812). Blue dash labels the slices through the dimer interface. (**b to e**) Detailed view of the four corresponding areas between the cross-sections from top to bottom illustrated as (**a**).

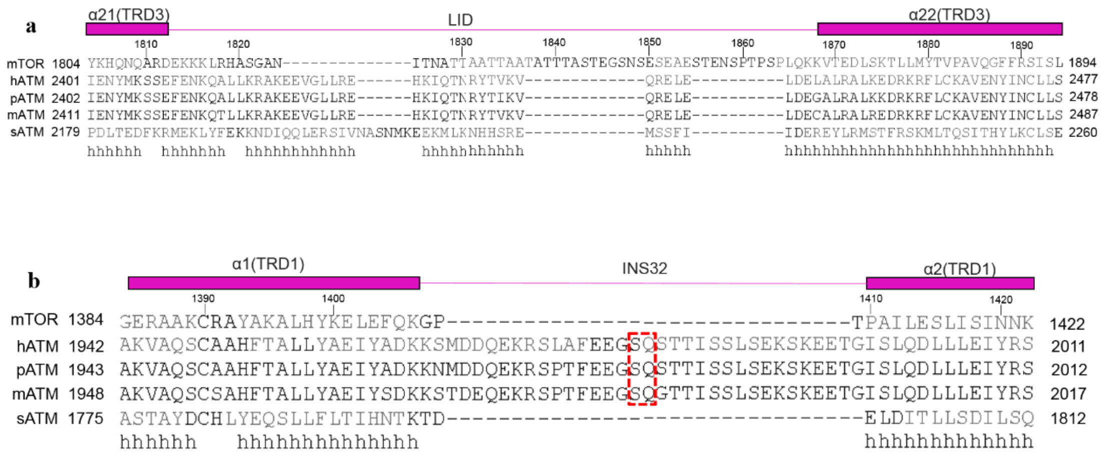

Supplementary Figure 8 | Conservation of the two disordered regions (LID and INS32) in ATM/Tel1 kinase. ATM sequences alignment for (**a**) LID region and (**b**) INS32 based on the mTOR structure [3], including the secondary structure restraints in the alignment with PROMALS3D [4]. ATM sequences from human (hATM), pig (pATM), mouse (mATM), and fission yeast (sATM) are used for the analysis.

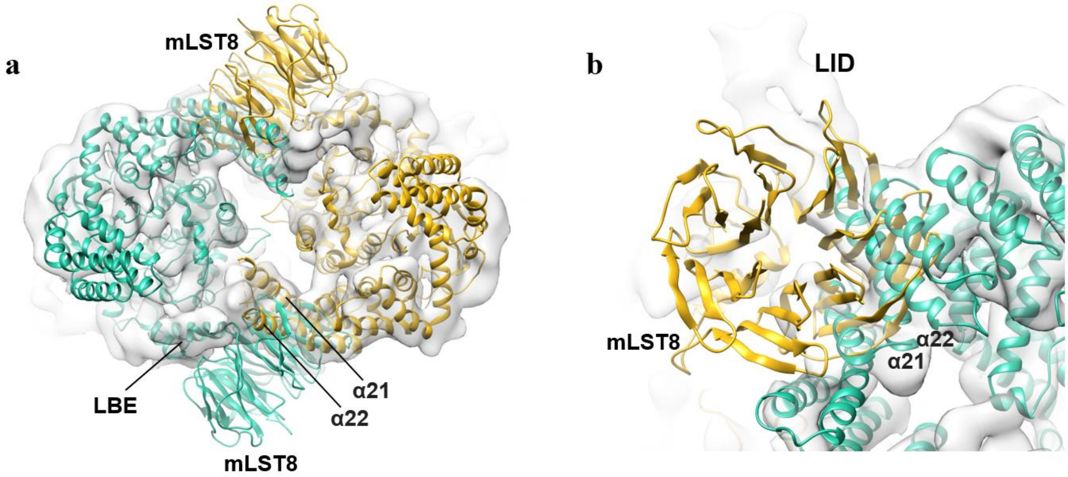

Supplementary Figure 9 | LID blocking binding of mLST8 to LBE in the mTOR dimer model. (**a**) Top view of ATM/Tel1dimer fitted by mTOR-mLST8 structure [3] showing LID evidently clashes with mLST8 in binding LBE. (**b**) Enlarged view of the overlapped region of mLST8 and LID.

Supplementary References

1. Radermacher, M. Three-dimensional reconstruction of single particles from random and nonrandom tilt series. *Journal of electron microscopy technique* **9**, 359-394 (1988).
2. Scheres, S. H. RELION: implementation of a Bayesian approach to cryo-EM structure determination. *Journal of structural biology* **180**, 519-530, doi:10.1016/j.jsb.2012.09.006 (2012).
3. Yang, H. *et al.* mTOR kinase structure, mechanism and regulation. *Nature* **497**, 217-223, doi:10.1038/nature12122 (2013).
4. Pei, J. & Grishin, N. V. PROMALS3D: multiple protein sequence alignment enhanced with evolutionary and three-dimensional structural information. *Methods in molecular biology* **1079**, 263-271, doi:10.1007/978-1-62703-646-7_17 (2014).
